# MicroRNAs Associated with Metastatic Prostate Cancer

**DOI:** 10.1371/journal.pone.0024950

**Published:** 2011-09-30

**Authors:** Akira Watahiki, Yuwei Wang, James Morris, Kristopher Dennis, Helena M. O'Dwyer, Martin Gleave, Peter W. Gout, Yuzhuo Wang

**Affiliations:** 1 Department of Experimental Therapeutics, British Columbia Cancer Agency, Vancouver, British Columbia, Canada; 2 Department of Radiation Oncology, British Columbia Cancer Agency, Vancouver, British Columbia, Canada; 3 Department of Diagnostic Imaging, British Columbia Cancer Agency, Vancouver, British Columbia, Canada; 4 Department of Urologic Sciences, Faculty of Medicine, University of British Columbia, Vancouver, British Columbia, Canada; 5 The Vancouver Prostate Centre, Vancouver General Hospital, Vancouver, British Columbia, Canada; Florida International University, United States of America

## Abstract

**Objective:**

Metastasis is the most common cause of death of prostate cancer patients. Identification of specific metastasis biomarkers and novel therapeutic targets is considered essential for improved prognosis and management of the disease. MicroRNAs (miRNAs) form a class of non-coding small RNA molecules considered to be key regulators of gene expression. Their dysregulation has been shown to play a role in cancer onset, progression and metastasis, and miRNAs represent a promising new class of cancer biomarkers. The objective of this study was to identify down- and up-regulated miRNAs in prostate cancer that could provide potential biomarkers and/or therapeutic targets for prostate cancer metastasis.

**Methods:**

Next generation sequencing technology was applied to identify differentially expressed miRNAs in a transplantable metastatic versus a non-metastatic prostate cancer xenograft line, both derived from one patient's primary cancer. The xenografts were developed via subrenal capsule grafting of cancer *tissue* into NOD/SCID mice, a methodology that tends to preserve properties of the original cancers (e.g., tumor heterogeneity, genetic profiles).

**Results:**

Differentially expressed known miRNAs, isomiRs and 36 novel miRNAs were identified. A number of these miRNAs (21/104) have previously been reported to show similar down- or up-regulation in prostate cancers relative to normal prostate tissue, and some of them (e.g., miR-16, miR-34a, miR-126*, miR-145, miR-205) have been linked to prostate cancer metastasis, supporting the validity of the analytical approach.

**Conclusions:**

The use of metastatic and non-metastatic prostate cancer subrenal capsule xenografts derived from one patient's cancer makes it likely that the differentially expressed miRNAs identified in this study include potential biomarkers and/or therapeutic targets for human prostate cancer metastasis.

## Introduction

Prostate cancer is the most common cancer in men and the second leading cause of cancer deaths in the United States [1]. While considerable advances have been made in the treatment of localized, organ-confined tumors, prostate cancer is currently incurable once it has progressed to metastasis, and most deaths from this disease are due to metastases that are highly resistant to conventional therapies. Currently, prostate-specific antigen (PSA) is a major serum biomarker used for the detection and monitoring of prostate cancer progression. However, the prognostic value of increased PSA levels is limited, since advanced prostate cancer can be associated with very low or normal PSA values. There is therefore an urgent need for new, more specific biomarkers which can be used to predict cancer progression on their own or in cooperation with a current biomarker such as PSA [2]. Furthermore, novel therapeutic targets associated with prostate cancer metastasis are urgently needed.

MicroRNAs (miRNAs) are small non-coding RNAs (17 to 27 nucleotides) that negatively regulate the expression of target genes by binding to 3′ untranslated regions (UTRs) of mRNAs and inhibiting translation or promoting mRNA degradation [3]. Recent studies have shown dysregulation of miRNAs in human tumors indicating a role for such molecules in cancer pathogenesis, including cancer onset, progression and metastasis [4], [5]. Thus far, only a small number of studies have investigated miRNA expression in prostate cancer, and only a few have dealt with metastasis of this disease. Differences in the expression profiles of miRNAs so far identified may have prognostic value for the various aspects of the disease and a better understanding of the role of miRNAs in the development and progression of prostate cancer is needed [6]. Further research may also lead to identification of new miRNAs that are specifically related to prostate cancer progression and metastasis. Such metastasis-associated miRNAs may serve as metastatic biomarkers and/or new targets for therapy of metastatic disease.

Studies aimed at identifying genetic factors with key roles in prostate cancer metastasis have been impeded by a lack of optimal experimental models. While xenograft models based on established cancer cell lines representing different stages of cancer progression can be useful for identifying mechanisms underlying metastasis, they do not adequately mimic clinical disease [7]. Efforts have therefore focused on use of patients' prostate cancer *tissues*. However, the typical heterogeneity of such tissues, consisting of both non-metastatic and potentially metastatic subpopulations, makes it difficult to identify factors such as genes that underlie the development of metastasis [8]. Moreover, it is difficult to obtain metastatic prostate cancer tissues from patients for experimental purposes, since they are not routinely or feasibly biopsied or resected from patients, and rapid autopsy programs are extremely expensive and difficult to manage. To overcome the above hurdles, we developed next generation patient-derived prostate cancer xenograft models, that more closely resemble the clinical situation, by using subrenal capsule grafting of patients' cancer tissue into immuno-deficient mice. This methodology favors retention of the properties of the original cancers [9]–[11]. Furthermore, it has been possible to establish transplantable, metastatic and non-metastatic prostate cancer sublines from heterogeneous xenografts [12], [13]. Use of metastatic and non-metastatic xenografts has already been effective in the identification of prostate cancer metastasis-associated genes [13].

Illumina's massively parallel DNA sequencing by synthesis technology is a widely-adopted next-generation sequencing platform. It supports parallel sequencing using a proprietary reversible terminator-based method that enables detection of single bases as they are incorporated into growing DNA strands. A fluorescently-labeled terminator is imaged as each dNTP is added and then cleaved to allow incorporation of the next base. Since all four reversible terminator-bound dNTPs are present during each sequencing cycle, natural competition minimizes incorporation bias, leading to true base-by-base sequencing [14].

In the present study, Illumina next generation sequencing technology was utilized to compare the miRNA profiles of a transplantable metastatic versus a non-metastatic prostate cancer xenograft line, both derived via subrenal capsule grafting [10]–[12] from one patient's primary cancer tissue. Differentially expressed known and novel miRNAs were found that may have specific roles in the metastasis of prostate cancer.

## Materials and Methods

### Patient-derived prostate cancer xenograft models

NOD/SCID mice used for xenografting were bred and maintained at the British Columbia Cancer Research Centre Animal Facility (Vancouver, Canada). All experimental protocols were approved by the University of British Columbia Animal Care Committee (A10-0100). A prostate cancer biopsy specimen was obtained at the BC Cancer Agency with the patient's written informed consent. Ethical approval was provided by the University of British Columbia - British Columbia Cancer Agency Research Ethics Board (UBC BCCA REB #H04-60131).

The establishment of transplantable prostate cancer tissue xenograft lines via subrenal capsule grafting has been described previously [9]. In the present study, a recently prepared metastatic prostate cancer xenograft line, LTL-313H [15], and a non-metastatic counterpart, LTL-313B (unpublished), were used that had been derived from different loci of one patient's prostate cancer biopsy sample (www.livingtumorlab.com). Both lines were PSA- and AR-positive as shown via immunohistochemistry ([15]; unpublished data). They were routinely maintained under renal capsules of male NOD/SCID mice supplemented with testosterone, as previously described [9]. The LTL-313H xenografts showed invasion of the mouse host kidney and cancer cells were detected in the lungs of the hosts after 3 months of grafting. In contrast, the LTL-313B xenografts showed no obvious invasion of the mouse kidney and did not show any distant metastases (data not shown).

### Small RNA library construction and cDNA sequencing

LTL-313H and LTL-313B xenograft tissues were collected and RNA was extracted using TRIzol (Invitrogen, Mississauga, ON, Canada) according to the manufacturer's instructions. The RNA was submitted to the Genome Sciences Centre at the British Columbia Cancer Agency (www.bcgsc.bc.ca) for small-RNA cDNA library construction and sequencing as previously described [16] with minor modifications. Each library had a specific index sequence in its 5′ adaptor, i.e. “ACATCGA” for the LTL-313H library and “CGTGATA” for the LTL-313B library; both libraries were mixed and the sequencing was run in one flow cell in the Illumina's platform.

### Small RNA mapping and differential expression detection

The 5′ indexed cDNA sequences were used to distinguish the origin of the RNAs. 3′ Adaptor sequences were removed from all reads and those remaining tags that were 16 to 27 nucleotides in length and expressed at a tag count of 2 or more in each library were used for further analysis. The trimmed sequences were mapped to miRBase 15 human stem-loop sequences (http://www.mirbase.org/) using the Novoalign (www.novocraft.com) program allowing up to 3 mismatches. Those that matched an miRBase sequence were then grouped as: 1) known mature miRNA and miRNA*, 2) putative miRNA*, not previously reported in the miRBase, 3) loop sequences and 4) sequences which matched the loop-sequence but did not have known mature sequences. The sequences matching known miRNAs were further clustered, based on their starting positions, and counted. The most abundant variation/starting position tags were used for comparison between libraries. Tag counts were normalized to the total counts of those sequences which matched the miRBase 15 stem-loop sequences and the two libraries were compared for differential expression of the sequences using the Fisher's exact test with Bonferroni's correction. Sequences were deemed significantly differentially expressed by the two libraries if the p-value was <0.001 and there was at least a 2–fold change in the sequences' normalized counts.

### Novel miRNA identification

The sequences that did not match known miRNA stem-loop sequences were filtered out with known transcripts sequences downloaded from UCSC [17]. To identify novel miRNA candidates amongst the remaining unmatched sequences, the miRanalyzer program [18] was used (http://web.bioinformatics.cicbiogune.es/microRNA/miRanalyser.php). The output candidates were checked one by one for homologies to known non-coding RNA, and non-homologous sequences were taken as novel miRNA candidates.

### miRNA target prediction and pathway analysis

The target genes for each differentially expressed miRNA were predicted using MicroCosm version 5 [19], [20] with a threshold of *p* = 0.001. As some of the genes were potentially regulated by both up-regulated and down-regulated miRNAs, we focused for further analysis on the genes that were potentially regulated only by up-regulated or down-regulated miRNAs. Identification of KEGG pathways associated with potential target genes was carried out using DAVID 6.7 (Database for Annotation, Visualization and Integrated Discovery, http://david.abcc.ncifcrf.gov/). In addition, we compared the target gene lists with gene expression data by microarray assay using the same xenograft tissues to identify those putative targets that might be regulated at the mRNA level.

### Microarray gene expression analysis

The RNA that was used for the miRNA sequencing library was also used for mRNA-based gene expression analysis using the Agilent's Human GE 44K platform at the Vancouver Prostate Centre Microarray Facility (www.mafpc.ca). All data are MIAME compliant and the raw data have been deposited in GEO (accession number GSE28029). The expression signal was transformed to z-score and calculated z-ratio and the mRNAs with more than 1.96 of z-ratio were dealt up-regulated and less than −1.96 as down-regulated [21]. The data were also filtered by Flag.

### Cell culturing

The 22Rv1 prostate cancer cell line was cultured in RPMI-1640 medium supplemented with 10% fetal bovine serum (FBS) at 37°C in a humidified atmosphere containing 5% CO_2_.

### miRNA precursor transfection

The precursor sequence of mir-486, shown in the miRBase, and a non-silencing negative control were subcloned into the pcDNA6.2-GW/EmGFP miR plasmid (Invitrogen). 22Rv1 cells were seeded in 12 well-plates at a density of 1.0×10^5^ cells per well, 24 hours in advance of transfection. Transfection was carried out using Lipofectamine 2000 (Invitrogen), following the manufacturer's instructions. The transfection efficiency was validated by GFP signal monitoring using an inverted fluorescence microscope system (Zeiss). To confirm increased levels of mature target miRNA, a portion of the transfected cells was used for validation by quantitative (qPCR). To this end, total RNA was extracted using a miRNeasy mini kit (Qiagen) and the quantity of RNA determined by nanodrop spectrophotometry (Thermo Scientific). Portions (25 ng) of each of the total RNA preparations were reverse-transcribed to cDNA using a Universal cDNA Synthesis Kit (Exiqon) following the manufacturer's instructions. The cDNA was diluted and mixed with microRNA LNA PCR primers and SYBR Green master mix (Exiqon). qPCR was carried out using an ABIPrism 7900HT (Applied Biosystems) following the manufacturer's instructions. The ΔΔCT was used for calculating the fold changes relative to the control and U6 was used as an endogenous control.

### MTT assay

The transfected cells were seeded in 96-well plates at a density of 1×10^4^ cells/well. MTT solution (20 µl of 5 mg/ml) was added to the cultures (200 µl volumes) for a 4 hr incubation at 37°C. Following removal of the culture medium, the remaining crystals were dissolved in DMSO and absorbance at 570 nm was measured.

### Migration/invasion assay

BioCoat Matrigel invasion chambers (BD Biosciences) were used to measure tissue invasiveness of cells. In the upper chambers, 1×10^5^ cells/well were plated in 0.50 ml of serum-free medium. In the lower chambers, 0.75 ml of medium/10% FBS was delivered. The chambers were incubated for 30 hr at 37°C in a humidified atmosphere with 5% CO_2_. The cells that remained in the upper chamber were removed and the transmigrated cells fixed in methanol and stained with crystal violet and stained cells were counted by microscopic analysis. Tumor cell invasion was expressed as the percentage of cells that had passed through the Matrigel-coated membranes relative to the number of cells that had passed through the uncoated membranes (invasion index). All assays were performed in triplicate.

## Results

### miRNA sequencing and annotation

Small RNAs were isolated from metastatic LTL-313H and non-metastatic LTL-313B prostate cancer tissue xenografts and processed to allow deep sequencing using the Illumina's platform. The reads with adaptor index sequences “ACATCGA” and “CGTGATA” were given LTL-313H origin and LTL-313B origin, respectively. More than 10 million total reads were obtained for each of the libraries. These reads were compared with the sequence data in the miRBase 15 microRNA Sequence Database. For the metastatic and non-metastatic prostate cancer tissue libraries, 3,445,642 and 2,272,677 tags, respectively, were fully mapped to human miRNA stem-loop sequences present in the miRBase database (Table 1). The completely matched reads were annotated, according to their position in the stem-loop structure. A shift of up to 2 bases in the starting and ending positions was allowed for sequences to be annotated as *isomers* of known mature miRNAs (isomiRs). The total numbers of known miRNAs plus miRNA*s in the metastatic and non-metastatic libraries were 447 and 509, respectively (Table 1). The most highly expressed miRNA (and isomiR) was the miR-148a with total counts of 270,801 and 763,877 reads per metastatic and non-metastatic libraries, respectively. When the isomiRs were grouped using the same starting position, the miR-148a remained the most abundant miRNA in the non-metastatic library with a total count of 846,468, whereas in the metastatic library miR-21 was most abundant with a total count of 310,102.

**Table 1:** Small-RNA library sequencing summary.

	Metastatic library	Non-metastatic library
index tag sequence	ACATCGA	CGTGATA
total cDNA reads	10,525,988	11,644,175
reads mapped to miRNA miRBase stem-loop sequences	2,272,677	3,445,642
unique sequences	1,875,353	2,941,722
most abundant miRNA	miR-148a (270,801)	miR-148a (763,877)
most abundant miRNA grouped at starting position, total	miR-21 (310,102)	miR-148a (846,468)
total known miRNA plus miRNA*	447	509

### miRNA and miRNA* expressions

In the miRBase, miRNAs derived from a precursor are designated miRNA, the predominantly expressed arm, and miRNA* the less-expressed, opposite arm. If both arms are similarly expressed, they are referred to as 5p and 3p arms. The expression of known miRNAs in the prostate cancer xenografts was in general higher than that of miRNA*s. In a number of cases, miRNA*s (as identified by miRBase) were more expressed than the corresponding miRNAs (Table 2). Thus miR-144* was substantially more expressed than miR-144 in both metastatic and non-metastatic libraries; similarly miR-126* was more expressed than miR-126, but only in the non-metastatic library. Differences were also found in the expression patterns of 3p and 5p arm miRNAs (Table 2). The 3p arms of miR-28 and miR-339 showed higher expressions than the corresponding 5p arms in the metastatic line, whereas they showed lower expressions than the corresponding 5p arms in the non-metastatic line. In the metastatic line, the miR-542 showed up-regulation of the 3p arm but down-regulation of the 5p arm. Fragments that were counterparts of known mature miRNAs, but that had not previously been reported to the miRBase database, were designated “putative novel miRNA*” species (Table 3). A total of 32 of such putative miRNA*s was observed showing at least two reads in one of the two libraries. Some of these miRNA*s also showed higher expression than their corresponding miRNAs, e.g., miR-1277* and miR-1307*.

**Table 2:** Levels^a^ of miRNA and miRNA* showing dysregulation in (i) miRNA∶miRNA* ratios in metastatic and non-metastatic xenografts and (ii) the levels of miRNA or miRNA* in the two xenograft lines.

	Expression level		
miRNA	metastatic	non-metastatic	fold change
miR-7	72.35	22.5	3.22
miR-7-1*	2.76	24.54	−8.88
miR-126	26005.24	1799.7	14.45
miR-126*	1099.03	3798.06	−3.46
miR-144	915.16	134.98	6.78
miR-144*	5429.24	418.37	12.98
miR-335	22.58	54.63	−2.42
miR-335*	65.9	91.74	1.39
miR-374a	1638.17	674.3	2.43
miR-374a*	954.79	825.93	1.16
miR-28-3p	317.5	188.15	1.69
miR-28-5p	184.78	1264.17	−6.84
miR-339-3p	65.43	102.55	−1.57
miR-339-5p	35.94	189.03	−5.26
miR-542-3p	100	26.88	3.72
miR-542-5p	0.92	12.27	−13.31

a Tag counts were normalized to the total counts of sequences that matched the miRBase 15 stem-loop sequences and are expressed as number per 1,000,000 tags.

**Table 3:** Putative miRNA*s, i.e. fragments complementary to known miRNAs but not reported in the miRBase.

			Expression levels			
putative miRNA* sequence	matched to miRNA stem-loop	starting position	putative miRNA*		miRNA	
			metastatic	non-metastatic	metastatic	non-metastatic
CTGTACAACCTTCTAGCTTTCC	hsa-let-7c†	56	4.15	1.17	2039.08	1584.38
TCAATAAATGTCTGTTGAAT	hsa-mir-95	15	0	0.88	44.70	973.18
TATACAACTTACTACTTTCC	hsa-mir-98	81	0	0.58	105.99	79.47
CGGGGCCGTAGCACTGTCTGA	hsa-mir-128-1	15	0.92	0	42.39	5.26
ATGTAGGGATGGAAGCCATGA	hsa-mir-135a-2	61	1.38	1.75	379.25	588.99
TGGAAACATTTCTGCACAAACT	hsa-mir-147b	12	0	1.17	1.38	0.29
GTCATTTTTGTGATGTTGCAG	hsa-mir-153-2	14	12.44	1.46	219.34	554.81
AGTGGTTCTTAACAGTTCAACA	hsa-mir-203	27	0	6.14	116.12	296.54
AGCCCCTGCCCACCGCACACTG	hsa-mir-210	28	1.38	1.17	13967.09	302.97
GCTCTGACGAGGTTGCACTACT	hsa-mir-301b	10	1.38	0.88	4.15	3.21
GTTCCTGCTGAACTGAGCCAGT	hsa-mir-3074	12	0	0.58	0.46	0.00
AGGGACTTTTGGGGGCAGATGTG	hsa-mir-365-1	16	1.38	1.17	16.59	22.50
GCGACGAGCCCCTCGCACAAACC	hsa-mir-375	5	0	0.88	6414.45	11574.16
TGTGTTGCATGTGTGTATATGT	hsa-mir-466	14	0.92	0	0	0
CAAAAGCAATCGCGGTTTTTGC	hsa-mir-548e	15	0	3.80	0.46	0
CAAAAACTGCAATTACTTTTGC	hsa-mir-548h-3	65	0	0.58	0	0
TTTGGTGCATATTTACTTTAGG	hsa-mir-559	57	2.76	0.58	0	0
ATCAAGGATCTTAAACTTTGCC	hsa-mir-561	26	3.23	1.46	0	0.88
TCGCGGTTTGTGCCAGATGAC	hsa-mir-579	25	0.92	0	0.46	2.34
ACAACCCTAGGAGAGGGTGCCATT	hsa-mir-652	20	0	1.17	11.06	16.65
GGACCTTCCCTGAACCAAGGA	hsa-mir-659	23	2.76	1.17	0	0
ACCTCCTGTGTGCATGGATT	hsa-mir-660	52	0	1.17	32.26	68.37
CGGCCCCACGCACCAGGGTAAG	hsa-mir-874	10	0.92	0	3.23	2.05
CGGGAACGTCGAGACTGGAGC	hsa-mir-1247	77	0.92	0.58	0	0.29
CTATCTTCTTTGCTCATCCTTG	hsa-mir-1255a	69	0	1.17	2.76	1.75
AGTTGGCATGGCTCAGTCCAAGT	hsa-mir-1269	25	0	5.55	9.22	7.89
TATATATATATATGTACGTATG	hsa-mir-1277	11	16.59	46.45	0	2.05
ATCTCACTTTGTTGCCCAGG	hsa-mir-1285-1	13	0.92	0	0	2.05
CTCTAGCCACAGATGCAGTGAT	hsa-mir-1287	55	0	2.05	0.92	7.89
GAGTGGGGCTTCGACCCTAACC	hsa-mir-1296	59	0	0.58	10.60	12.27
CCACCTCCCCTGCAAACGTC	hsa-mir-1306	15	1.38	1.75	0	0.58
TCGACCGGACCTCGACCGGCTCG	hsa-mir-1307	41	82.48	126.50	11.98	26.59

† putative let-7c* sequence is not identical to the let-7c* in the miRBase but shows complementarity to let-7c.

### Novel miRNA candidates

One advantage of utilizing a sequencing approach for miRNA profiling is the opportunity to identify novel miRNAs or miRNA*s. To this end, we used an miRanalyzer, a microRNA detection and analysis tool [18], in combination with homology searches to identify known transcripts, including non-coding RNAs (e.g., rRNA, tRNA, etc.). Using miPred software to distinguish real pre-miRNAs from other hairpin sequences with similar stem-loops [22], we identified 36 novel miRNA candidates. Their sequences, chromosome locations and number of reads in the metastatic and non-metastatic libraries are presented in Table 4 and Table S1. Comparative analysis of the two libraries showed significant differential expression of some of these novel miRNAs, including down-regulated miR-5680-3p and miR-5681a-3p.

**Table 4:** Novel miRNA candidates.

			reads in	
ID	Sequence	chromosomal location	metastatic	non-metastatic
hsa-miR-5680-3p	GAGAAATGCTGGACTAATCTGC	8q22.3	28	318
hsa-miR-5681a-3p	AGAAAGGGTGGCAATACCTCTT	8q21.11	4	66
hsa-miR-5682-3p	GTAGCACCTTGCAGGATAAGGT	3q13.33	2	29
hsa-miR-548aw-5p	GTGCAAAAGTCATCACGGTT	9q34.13		26
hsa-miR-5683-5p	TACAGATGCAGATTCTCTGACTTC	6p25.1	25	22
hsa-miR-5684-5p	AACTCTAGCCTGAGCAACAG	19p13.13	2	2
hsa-miR-548ax-5p	AGAAGTAATTGCGGTTTTGCCA	Xp22.2		12
hsa-mir-5685-5p	ACAGCCCAGCAGTTATCACGGG	6p12.1		9
hsa-miR-5692c-3p	AATAATATCACAGTAGGTGTAC	5q31.1		8
		7q21.3		
hsa-miR-5686-5p	TATCGTATCGTATTGTATTGT	10q24.1	8	
hsa-miR-5687-3p	TTAGAACGTTTTAGGGTCAAAT	5q11.2		6
hsa-miR-5688-3p	TAACAAACACCTGTAAAACAGC	3p12.1	5	
hsa-miR-5681b-5p	AGGTATTGCCACCCTTTCTAGT	8q21.11	4	
hsa-miR-548at-5p	AAAAGTTATTGCGGTTTTGGC	17q21.31		4
hsa-miR-5689-5p	AGCATACACCTGTAGTCCTAGA	6p24.3		4
hsa-miR-5690-5p	TCAGCTACTACCTCTATTAGG	6p21.31	3	
hsa-miR-5691-5p	TTGCTCTGAGCTCCGAGAAAGC	11p15.4		3
hsa-miR-5692a-5p	CAAATAATACCACAGTGGGTGT	7q21.3		3
		8p23.1		
hsa-miR-4666b-5p	TTGCATGTCAGATTGTAATTCCC	Xp21.2		3
hsa-miR-5693-3p	GCAGTGGCTCTGAAATGAACTC	13q14.3	2	
hsa-miR-5694-5p	CAGATCATGGGACTGTCTCAG	14q23.3	2	
hsa-miR-5695-3p	ACTCCAAGAAGAATCTAGACAG	19p13.13	2	
hsa-miR-5696-5p	CTCATTTAAGTAGTCTGATGCC	2q11.2	2	
hsa-miR-5697-5p	TCAAGTAGTTTCATGATAAAGG	1p36.22		2
hsa-miR-5698-5p	TGGGGGAGTGCAGTGATTGTGG	1q21.3		2
hsa-miR-5699-3p	TCCTGTCTTTCCTTGTTGGAGC	10p15.3		2
hsa-miR-5700-5p	TAATGCATTAAATTATTGAAGG	12q22		2
hsa-miR-5701-5p	TTATTGTCACGTTCTGATT	15q11.2		2
hsa-miR-5702-3p	TGAGTCAGCAACATATCCCATG	2q36.3		2
hsa-miR-5703-3p	AGGAGAAGTCGGGAAGGT	2q36.3		2
hsa-miR-5692b-5p	AATAATATCACAGTAGGTGT	21q22.3		2
hsa-miR-5704-5p	TTAGGCCATCATCCCATTATGC	3q22.1		2
hsa-miR-5705-3p	TGTTTCGGGGCTCATGGCCTGTG	4q22.1		2
hsa-miR-5706-5p	TTCTGGATAACATGCTGAAGCT	5q23.1		2
hsa-miR-5707-5p	ACGTTTGAATGCTGTACAAGGC	7q36.3		2
hsa-miR-5708-5p	ATGAGCGACTGTGCCTGACC	8q21.13		2

### Potential metastasis-associated miRNAs

Comparative analysis of the metastatic and non-metastatic xenograft miRNA libraries revealed a total of 104 differentially expressed miRNAs or miRNA*s with 55 down-regulated and 49 up-regulated in the metastatic line (Table 5,6,7). Of the down-regulated miRNAs, 24 miRNAs showed a >5-fold decrease, including four miRNAs, i.e. miR-205, miR-503, miR-708 and miR-2115*, which were undetectable in the metastatic line. Two miRNAs, i.e. miR-24-2* and miR-101*, showed increased expression in a one-base-shift form. A one-base-shift form of miR-203 showed some increased expression in the metastatic line relative to reference miR-203, whereas in the non-metastatic line it showed a lower expression. Of the up-regulated miRNAs, 23 miRNAs showed a >5-fold change in normalized counts. One-base-shift forms of miR-9*, miR-148b* and miR-1246 showed higher expression than the reference forms in both metastatic and non-metastatic lines.

**Table 5:** miRNAs down-regulated in the metastatic library§.

				references		
				prostate cancer	metastasis in	
miRNA	metastatic	non-metastatic	fold change		prostate cancer	other types of cancer
miR-7-1*	2.76	24.54	−8.88			
miR-15b	489.84	2660.69	−5.43	[28] #		
miR-16	999.03	17741.34	−17.76	[23]–[25], [38] #	[25]	
miR-24	1046.96	8903.54	−8.50	[26]–[28], [76] #		[61]
miR-24-2*†	5.07	22.50	−4.44			
miR-26b	1964.89	8991.18	−4.58	[28] #		
miR-28-5p	184.78	1264.17	−6.84			
miR-29a	446.06	13028.23	−29.21	[26], [28], [29]		
miR-29c	299.99	1643.68	−5.48			
miR-33b	22.12	62.81	−2.84			
miR-34a	76.03	194.87	−2.56	[77], [26] #	[33]	[78]
miR-95	57.60	1128.90	−19.60	[26] #		
miR-101*†	4.61	105.76	−22.95			
miR-106b	906.41	6545.23	−7.22	[30] #		
miR-126*	1099.03	3798.06	−3.46	[28] #	[34]	
miR-145	103.68	297.71	−2.87	[23], [24], [27], [29], [30]	[35]	[61]
miR-146b-5p	1335.88	2765.57	−2.07	[26] #		[79], [61] #
miR-185	84.79	448.76	−5.29			[60]
miR-186	1666.28	5838.21	−3.50			[61] #
miR-188-5p	0.92	9.64	−10.46			
miR-191	1822.50	12911.66	−7.08	[26] #		
miR-193a-3p	35.94	196.91	−5.48			
miR-195	116.58	560.65	−4.81	[23], [26] #		
miR-196a	33.64	70.99	−2.11	[26] #		
miR-200b*	146.54	499.30	−3.41			
miR-200c*	12.44	54.34	−4.37			
miR-203	147.92	434.73	−2.94	[26] #		[61]
miR-203‡	224.41	334.81	−1.49	[26] #		
miR-205	0.00	31.26	N/A	[24], [31], [32]	[36]	

† most abundant miRNA started one base upstream from the mature form of miRBase.

‡ most abundant miRNA started one base downstream from the mature form of miRBase.

§ Differential expressions all had a >2-fold change and a p<0.001 and are considered statistically significant.

# Results from the literature that do not match the down- or up-regulation found in the present study.

**Table 6:** miRNAs down-regulated in the metastatic library§(Continued from Table 5).

				references		
				prostate cancer	metastasis in	
miRNA	metastatic	non-metastatic	fold change		prostate cancer	other types of cancer
miR-324-5p	27.19	61.94	−2.28			
miR-331-3p	2.76	14.32	−5.18	[80]		
miR-335	22.58	54.63	−2.42			[62]
miR-339-5p	35.94	189.03	−5.26			
miR-342-3p	61.75	247.46	−4.01			
miR-361-5p	103.68	259.73	−2.51			
miR-363	1298.10	8476.40	−6.53			
miR-424	22.12	76.25	−3.45	[28]		
miR-425	1869.04	10985.75	−5.88			
miR-454	10.60	32.72	−3.09			
miR-497	164.05	373.67	−2.28	[23]		
miR-503	0.00	7.30	N/A	[23] #		
miR-542-5p	0.92	12.27	−13.31			
miR-556-5p	1.84	13.15	−7.13	[26] #		
miR-582-5p	49.77	203.93	−4.10			
miR-590-5p	12.44	213.86	−17.19			
miR-627	2.30	93.20	−40.45			
miR-651	11.06	54.63	−4.94			
miR-652	12.90	33.89	−2.63			
miR-660	51.15	110.14	−2.15			
miR-664	8.76	30.97	−3.54			
miR-708	0.00	42.36	N/A			
miR-1180	3.69	15.48	−4.20			
miR-1269	11.06	42.66	−3.86			
miR-1287	1.84	12.85	−6.97			
miR-2115*	0.00	7.89	N/A			
miR-3065-5p	14.75	117.74	−7.98			

§ Differential expressions all had a >2-fold change and a p<0.001 and are considered statistically significant.

# Results from the literature that do not match the down- or up-regulation found in the present study.

**Table 7:** miRNAs up-regulated in the metastatic library§.

				references		
				prostate cancer	metastasis in	
miRNA	metastatic	non-metastatic	fold change		prostate cancer	other types of cancer
let-7d	671.86	322.84	2.08	[26]		
let-7g	8690.38	3448.64	2.52	[23] #		
let-7g*	42.39	3.21	13.19			
let-7i	851.11	400.55	2.12	[26]		[61]
miR-7	72.35	22.50	3.22			
miR-9	20121.64	1386.00	14.52			[65],[61] #
miR-9*‡	352.98	35.64	9.90			
miR-17	12228.92	5600.39	2.18			
miR-18a	1023.45	142.57	7.18			
miR-18b	90.78	26.59	3.41			
miR-20b*	89.40	7.60	11.77			
miR-27a	1568.59	721.34	2.17	[26], [28] #		
miR-27b	1406.39	425.38	3.31	[28] #		
miR-30a	30144.22	10956.83	2.75			[61] #
miR-30a*	1300.40	532.90	2.44			
miR-31	80.64	3.80	21.23	[30], [24] #		[67], [81]
miR-34c-5p	2190.22	51.13	42.84			
miR-99a	1017.92	330.43	3.08	[23] #		
miR-106a	2790.65	845.51	3.30	[26],[28] [38] #		
miR-125b	1699.46	287.19	5.92	[38]–[40], [23], [24], [27], [29] #		[61]
miR-125b-2*	15.67	1.46	10.73	[57]		
miR-126	26005.24	1799.70	14.45	[54] #		
miR-128	81.10	15.48	5.24	[26] #		
miR-136	40.55	7.89	5.14			
miR-138	38.25	1.46	26.18			
miR-140-5p	2458.87	723.97	3.40			
miR-142-5p	104.60	26.00	4.02			[61]
miR-144	915.16	134.98	6.78			
miR-144*	5429.24	418.37	12.98			
miR-148b*†	258.97	111.31	2.33			
miR-151-3p	4377.22	1866.89	2.34			[66]
miR-152	2662.55	582.56	4.57	[28] #		
miR-181a-2*	19.81	0.88	22.61			
miR-200a	7217.64	1066.09	6.77	[58] #		
miR-210	16686.78	347.38	48.04	[23]		
miR-218	140.55	70.12	2.00	[25],[26] #		
miR-223*	16.13	0.29	55.20			
miR-301a	60.83	21.62	2.81		[37]	
miR-340*	46.08	16.36	2.82			
miR-374a	1638.17	674.30	2.43			
miR-379	104.14	35.94	2.90			
miR-449a	18.43	1.17	15.77	[59] #		
miR-450a	191.24	24.83	7.70			[61]
miR-451	4504.40	2197.62	2.05			
miR-486-3p	9.22	0.88	10.52			
miR-486-5p	430.86	54.93	7.84			
miR-542-3p	100.00	26.88	3.72			
miR-744*	17.05	3.80	4.49			
miR-1246‡	95.39	22.50	4.24			

† most abundant miRNA started one base upstream from the mature form of miRBase.

‡ most abundant miRNA started one base downstream from the mature form of miRBase.

§ Differential expressions all had a >2-fold change and a p<0.001 and are considered statistically significant.

# Results from the literature that do not match the down- or up-regulation found in the present study.

Some of the differentially expressed miRNAs have previously been associated with prostate cancer, prostate cancer metastasis or metastasis of other types of cancer (Table 5,6,7). Of the down-regulated miRNAs a number have been reported to be down-regulated in prostate cancer relative to benign prostate tissues, i.e. miR-16 [23]–[25], miR-24 [26]–[28], miR-29a [26], miR-145 [23], [24], [27], [29], [30], and miR-205 [24], [31], [32]. The down-regulation of miR-16 [25], miR-34a [33], miR-126* [34], miR-145 [35] and miR-205 [36] correlated with the development of prostate cancer metastasis. Of the up-regulated miRNAs (in the metastatic library), miR-210 has been reported to be up-regulated in prostate carcinomas relative to BPH samples [23] and miR-301 has been linked to prostate cancer metastasis [37]. In some cases, miRNAs that were found to be up-regulated in the present study have been reported to be either up-regulated in prostate carcinomas compared to normal prostate tissue [38]–[40], or down-regulated [23], [24], [27]–[29]. Furthermore, some of the differentially expressed miRNAs have been reported to play a role in the metastasis of other types of cancer, for example, the up-regulated miRNAs, let-7i, miR-9, miR-30a, miR-125b, miR-142-5p, miR-151-3p, miR-450a and the down-regulated miRNAs, miR-24, mir-145, miR-146b-5p, miR-185, miR-186, miR-203 and miR-335.

### Putative target genes for differentially expressed miRNAs

As a first step in the identification of miRNAs with potential significance in the metastatic process, we identified putative target genes for each of the differentially expressed miRNAs using Microcosm analysis, a target prediction program with a specific algorithm and coverage of miRNA, including varieties in star arms; a threshold *p*-value = 0.001 was maintained to get more reliable target identification (Microcosm) [41]. Putative target genes were identified for 49 out of 55 down-regulated miRNAs and for 47 out of 49 up-regulated miRNAs (Table S2); they were annotated using the DAVID program. The putative target genes of the down-regulated miRNAs were associated with a variety of KEGG pathways including “Fc gamma R-mediated phagocytosis” and “ECM-receptor interaction (Table S3). For the putative target genes of the up-regulated miRNAs, pathways such as “Pathways in cancer”, “Focal adhesion” and “Purine metabolism” were noted.

### Comparison of putative miRNA target genes with genes differentially expressed in the metastatic and non-metastatic xenograft lines

Although miRNAs are thought to alter protein levels, they have in some cases been shown to also affect mRNA levels [3]. In view of this, the two xenograft lines were examined for differential mRNA expression. As shown in the Table S4, 622 mRNAs were down-regulated and 348 mRNAs were up-regulated in the metastatic line. Some of the genes identified by differential mRNA expression were potentially targeted by both up- and down-regulated miRNAs. In view of this, further analysis was restricted to genes that were potentially targeted by either up-regulated or down-regulated miRNAs. The group that showed up-regulated mRNAs associated with only down-regulated miRNAs consisted of 85 mRNAs; the group that showed down-regulated mRNAs associated with only up-regulated miRNAs consisted of 58 mRNAs. Among the mRNAs up-regulated in the metastatic line, some have been reported to have a role in tissue invasion and/or metastasis of a variety of cancer cells, including mRNA expressed by *FSCN1* [42], *VEGFA* [43] *FGFR1* [44], *ADAMTS1* [45], *CCL2* [46] and *VIM* [47] genes. Similarly, mRNAs down-regulated in the metastatic line, including mRNAs expressed by the *CTGF* [48] and *SERPINB5* [49] genes, have been found to be down-regulated in various metastatic cancers, attesting to the reliability of our analyses (Table S5).

### Increased levels of mature miR-486 in transfected cells

At 24 hours following transfection of 22Rv1 cells with pcDNA6.2-GW/EmGFP-mir486 or pcDNA6.2-GW/EmGFP-control sequence, more than 90% of the cells were found to be GFP positive. The mir-486 precursor had been properly processed to the mature miR-486 form as indicated by qPCR. Relative to the control, the expression levels of both miR-486-5p and -3p arms were 11.6 fold higher, indicating that the majority of the cells expressed elevated miR-486 levels.

### Invasiveness of miR-486-transfected 22Rv1 cells

**Figure 1:**
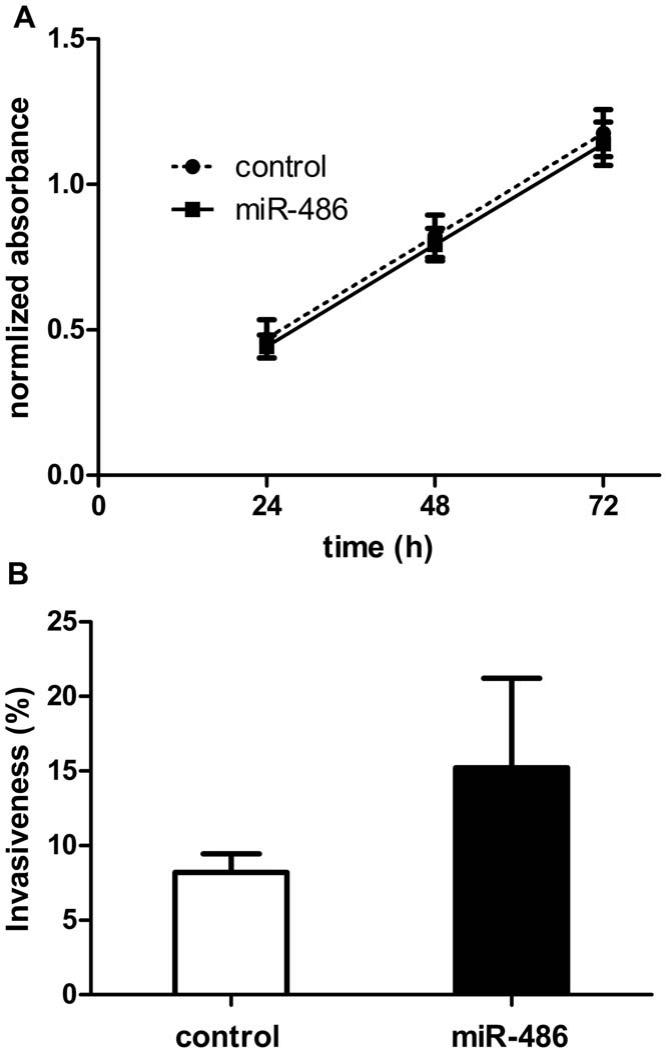
Effects of overexpression of miR-486 on proliferation and tissue invasiveness of 22Rv1 human prostate cancer cells. **A**) As indicated by MTT assay, there was no significant difference between the growth of miR-486-transfected cells and control cells over a 72-hr period. **B**) Tissue invasiveness, as measured by Matrigel invasion, of miR-486 transfected cells and control cells. Data are expressed as percent invasiveness ± S.D. and show increased invasiveness of miR-486-transfected cells (85% ; *p* = 0.08).

The proliferation rate of miR-486-transfected and control sequence-transfected cells was similar as indicated by the MTT assay (Fig. 1A). However, the miR-486-transfected cells showed an increase of about 85% in tissue invasiveness relative to the control cells (Fig. 1B). Although this result has borderline significance (*p* = 0.08), it indicates that increased expression of miR-486 enhances tissue invasiveness.

## Discussion

MicroRNAs have been implicated in the regulation of gene expression at the post-transcriptional level in almost every biological event, and there is an increasing body of evidence that altered expressions of specific miRNAs are involved in the development and progression of cancers [4], [5]. Using next generation sequencing for small RNA identification, the present study was aimed at identifying differentially expressed known and novel miRNAs in metastatic versus non-metastatic prostate cancer xenografts that could play a role in the progression of prostate cancer to the metastatic form. The transplantable cancer lines that were used appear to be highly suitable for the purpose since they had been derived from one patient's cancer and thus possessed a common genetic background. Furthermore, they had been developed via subrenal capsule grafting of cancer *tissue* into NOD/SCID mice, a methodology that tends to preserve important properties of the original cancers (e.g., tumor heterogeneity, genetic profiles) [9]–[11], [50]. As well, the maintenance of the tumor lines in the same type of graft site (under the kidney capsule) ensured that their growth was not markedly influenced by micro-environmental differences that can have an important impact on cancer development [51]. Similarly, the same type of graft site would minimize differences in miRNA production by host cells present in the xenografts. Although it has been shown that xenografting can alter the expression of miRNAs [52], our study focused primarily on differences in miRNAs between matched samples and these differences are therefore likely to be real. Taken together, the data obtained in this study should be useful for the delineation of miRNAs with oncogenic properties that are involved in the development of prostate cancer metastasis.

The highest reads in the two RNA libraries were observed for miR-148a. The expression of this miRNA is androgen-inducible in LNCaP cells [53]. This suggests that the relatively high expression of miR-148a found in the two libraries is a result of the testosterone supplementation of the animals.

Of the 104 miRNAs that were found to be down- or up-regulated in the metastatic prostate cancer xenografts, relative to their non-metastatic counterparts, 39 had previously been reported to be involved in prostate cancer (Table 5,6,7). These reports were mostly based on comparisons of miRNA expressions in prostate cancer tissues versus normal prostate tissues without defining the metastatic ability of the malignant samples. It is of interest that 21 of the 39 miRNAs showed down- or up-regulations in the metastatic xenografts which matched those reported for the prostate cancer tissues (relative to benign tissues), suggesting that these prostate cancer tissues may have had metastatic ability. Of the miRNAs found to be down-regulated in the metastatic xenografts, miR-16, showing a >17-fold decrease in expression, has been reported to be down-regulated in prostate cancer [23], [24] and to have a metastasis-suppressing function. Moreover, metastatic prostate tumor growth *in vivo* could be inhibited by systemic delivery of synthetic miRNA-16 [25]. The reduced expression of miR-34a in the metastatic xenograft line is consistent with its reported inhibition of prostate cancer metastasis [33]. The lower expression of miR-126* is in agreement with reports that this miRNA is down-regulated in prostate cancer metastasis [34] and that ectopic expression of miR-126* inhibited the migration and invasiveness of prostate cancer cells [34]. The latter being an example of a miRNA* playing a role as a tumor suppressor. Interestingly, miR-126, a miRNA reported as down-regulated in prostate cancer relative to normal prostate tissue [54], was up-regulated in the metastatic xenograft line. In this context it is of interest that whereas in LNCaP cells, the antagomir of miR-126 did not affect cell migration, the antagomir of miR-126* induced cell migration with up-regulation of prostein [34], suggesting that miR-126* affects cell migration more than miR-126. This raises the possibility that alternative strand selection as a mechanism for changing the expression of either arm is involved in the development of cancer or cancer metastasis.

The down-regulation of miR-145 in the metastatic xenograft line is in agreement with many reports identifying it as down-regulated in prostate tumors [23], [24], [27], [29], [30]. As well, a role for miR-145 in prostate cancer metastasis is suggested by its down-regulation observed in clinical samples of metastatic prostate cancer relative to localized high grade prostate cancer [35]; furthermore, miR-145 is considered a putative tumor suppressor in colon cancer cells [55] and can reduce breast cancer cell motility [56]. The expression of miR-205 was also reported to be down-regulated in prostate cancer cells, and ectopically expressed miR-205 showed a tumor-suppressive effect, including reduction of cell migration and tissue invasion [36].

Of the miRNAs found to be up-regulated in the metastatic xenografts, miR-125b has been reported to be up-regulated in prostate cancer and shown to be oncogenic [24], [29], [40]; other studies, however, have reported miR-125b to be down-regulated in prostate cancer [23], [24], [27]–[29]. miR-125b-2, a component of the miR-125b cluster, has been identified as part of an androgen receptor-mediated transcriptional network [57]. In a number of single studies, miRNAs such as let-7d [26], let-7i [26] and miR-210 [23] were also found to be up-regulated in prostate cancer, in contrast to let-7g [23], miR-27b [28], miR-99a [23], miR-126 [54], miR-128 [26], miR-152 [28], miR-200a [58] and miR-449a [59] which were down-regulated in prostate cancer samples. Both up- and down-regulation in prostate cancer was reported for a number of miRNAs. The reason for these discrepancies is not clear. Our finding indicating that upregulation of miR-486 is coupled to increased tissue invasiveness, as found with 22Rv1 human prostate cancer cells (Fig. 1b), supports the biological significance of the present study.

It is apparent from the above discussion that a number of the differentially expressed miRNAs identified in this study probably have a significant role in prostate cancer metastasis. Thus some of the miRNAs have already been linked to this phenomenon, in particular down-regulated miRNAs such as miR-16, miR-34a, miR-126*, miR-145 and miR-205, supporting the validity of our analytical approach. However, the prostate cancer xenografts did not show significant differential expression for miRNAs such as miR-221, whose down-regulation in a study using prostate cancer samples from a large number of patients was reported to be a hallmark in human prostate cancer metastasis [37]. This deficiency likely stems from the tumor heterogeneity of prostate cancers and illustrates the need for using a larger number of matched metastatic and non-metastatic xenografts and also clinical samples.

The present study has also identified differentially expressed miRNAs that have not previously been linked to prostate cancer, but to metastasis of other types of cancer (Table 5,6,7). Of the miRNAs down-regulated in the metastatic xenografts, miR-185 has been shown to suppress growth and progression of certain human cancers (e.g., breast, ovary) by targeting the *Six1* oncogene which regulates c-myc expression [60]. The miR146b-5p [61] and miR-335 [62] miRNAs have been shown to be metastasis suppressors in breast and colon cancers, facilitating the metastatic phenotype at reduced levels [63]. Of the up-regulated miRNAs in the metastatic line, miR-9 has been reported to target E-cadherin [64] and CDH1, the E-cadherin-encoding messenger RNA [65]; overexpression of miR-9 in non-metastatic breast tumor cells enables such cells to form pulmonary micrometastases in mice [65]. The miR-30a, miR-142-5p and miR-450a have roles in metastatic breast and colon cancer [61] and the miR-151-3p can enhance hepatocellular carcinoma cell mobility [66]. The upregulation of miR-31 is consistent with its ability to induce migration and tissue invasion of colon cancer cells via targeting of T-cell lymphoma invasion and metastasis 1 (TIAM1) [67]. It appears likely that these miRNAs also have a critical role in the development of prostate cancer metastasis on the basis of their role in the metastasis of other cancers, but further validation is needed.

The identification of novel putative miRNAs (Table 4) is of major interest for follow-up studies. In advanced prostate cancer, DNA copy number gain is commonly observed in the chromosome 8q arm [68], and the LTL-313 xenograft lines that were used in the present study also show an 8q arm copy number gain (data not shown). The finding that five of the 46 novel miRNAs were located on chromosome 8q, including the most abundantly expressed candidates (Table 4), suggests that there is a correlation between tissue-specific expression of an miRNA and its DNA copy number.

As found in the present study, some of the miRNA*s (identified by miRBase) are more highly expressed than their corresponding miRNAs (Table 2). This is likely a result of cancer-induced changes in miRNA processing and stability, including strand selection [69]. The latter is thought to normally involve an RNA-Induced Silencing Complex (RISC), a multiprotein complex that can incorporate one strand of an miRNA for subsequent silencing of the complementary mRNA [70]. Switching of a strand should lead to activation/inhibition of a different set of target genes and may underlie oncogenic properties of miRNA*s. There are several reports of miRNA* contributions in cancer progression [34], [71], [72].

The occurrence of miRNA isoforms (isomiRs) in the present study has also been observed in other studies using miRNA deep sequencing [16]. The variability in isomiR expression during Drosophila melanogaster development, generally thought to result from inexact Dicer processing and RNA editing, may not be arbitrary and in fact be regulated and biologically meaningful [73]. At present, the contribution of isomiRs in target recognition is not clear. However, it appears likely that the seed sequence of mature miRNA, i.e. the first 2–8 nucleotides at the 5′ end, is a key of the miRNA's target recognition and a variation of the 5′ end could readily alter the group of target genes of the miRNA. Likewise, the role of isomiRs in cancer has to be elucidated. Lee et al. [74] have constructed a database cataloguing an entire repertoire of miRNA sequences that can be useful for showing isomiR expression pattern differences in various cell types and conditions. The role of the differentially expressed miRNAs, including miRNA*, isomiR and novel miRNA (candidates), in the development of metastatic prostate cancer remains to be shown. Use of patient-derived prostate cancer xenograft models in conjunction with clinical sample analysis and *in vitro* models may bring unique perspectives to translational research.

It is of interest that many of the genes associated with the differentially expressed mRNAs in the xenografts were found to be identical to predicted target genes of differentially expressed miRNAs, and that they were related to cancer and metastasis, e.g., *FSCN1* [42], *CCL2* [46], *ADAMTS1* [45], *FGFR1* [44], *CTGF* [48] and *SERPINB5* [49]. However, while an miRNA potentially has hundreds of target genes, relatively few targets have been experimentally validated and few miRNA loss-of-function phenotypes have been assigned [75]. More research is required into the effect of specifically inhibiting/enhancing the function of miRNAs on the activity of their putative target genes, gene translation and the various stages of cancer development.

In summary, we have utilized next generation sequencing to identify differentially expressed known and novel miRNAs in a pair of metastatic and non-metastatic prostate cancer xenografts derived from one patient's primary cancer. The use of xenografts generated by subrenal capsule grafting of cancer tissue, a technique that tends to preserve properties of the original cancers, coupled to the finding that a substantial number of the differentially expressed genes have previously been linked to metastasis of prostate cancer or other types of cancer, makes it likely that the identified miRNAs include potential biomarkers and/or therapeutic targets for prostate cancer metastasis.

## Supporting Information

Table S1 **Novel miRNA candidates.**
(XLS)

Table S2 **List of target genes of up-regulated and down-regulated miRNAs as predicted via the Microcosm database.**
(XLS)

Table S3 **Enriched KEGG pathways using the DAVID analysis of up-regulated and down-regulated miRNA target genes.**
(XLS)

Table S4 **List of up/down-regulated mRNAs identified via microarray analysis.**
(XLS)

Table S5 **Lists of genes associated with (i) up-regulated mRNAs/down-regulated miRNAs and (ii) down-regulated mRNAs/up-regulated miRNAs.**
(XLS)
